# Current News

**Published:** 1894-09

**Authors:** 


### Current News.

### MISSOURI STATE DENTAL ASSOCIATION.

The Thirtieth Annual Meeting of the Missouri State Dental Association was held at Excelsior Springs, July 10 to 13, 1894. The following officers for the ensuing year were elected:

Dr. J. T. Fry, Moberly, President; Dr. D. F. Orr, Liberty, First Vice-President; Dr. W. L. Reed, Mexico, Second Vice-President; Dr. W. M. Carter, Sedalia, Corresponding Secretary; Dr. S. C. A. Ruby, Clinton, Recording Secretary; Dr. James A. Price, Savannah, Treasurer; Dr. James A. Price, Savannah, Committee on Law.

The next meeting will be held at Pertle Springs, beginning on the first Tuesday after the Fourth of July, 1895, and continuing in session four days.

W. M. Carter,
*Corresponding Secretary.*

### FIRST DISTRICT DENTAL SOCIETY OF ILLINOIS.

The First District Dental Society of Illinois will hold its Annual Meeting September 11 and 12.

A thoroughly practical programme has been prepared,—"Papers" and "Clinics,"—and the meeting will prove interesting and instructive.

O. M. Daymude, Monmouth, Ill.,
*President.*
W. O. Butler, La Harpe, Ill.,
*Secretary.*

